# Unequal recovery of health facility visits in hospitals and township health centres after COVID-19 disruption in Henan Province, China, 2014–2024: a time-series study

**DOI:** 10.3389/fpubh.2026.1916015

**Published:** 2026-10-28

**Authors:** Xin Tan, Yu Hua, Yanwei Ma, Yulin Shi

**Affiliations:** 1 Department of Cardiology II, The First People’s Hospital of Nanyang, Nanyang, Henan, China; 2 Department of Medical Oncology I, The First People’s Hospital of Nanyang, Nanyang, Henan, China; 3 Department of Cardiology I, The First People’s Hospital of Nanyang, Nanyang, Henan, China; 4 Center for Evidence-Based Medicine, Beijing Institute of Respiratory Diseases, Beijing Chao-Yang Hospital, Capital Medical University, Beijing, China

**Keywords:** China, counterfactual prediction, COVID-19, health facility visits, long short-term memory

## Abstract

**Background:**

The COVID-19 pandemic disrupted health service utilisation worldwide. Its long-term effect remains uncertain, especially after the major adjustment of China’s COVID-19 control policies at the end of 2022. Evidence is also limited on whether recovery differed between hospitals and township health centres (THCs). The research aimed to evaluate the impact of the COVID-19 pandemic and its ending on health facility visits in hospitals and THCs in Henan Province from 2014 to 2024.

**Methods:**

Monthly aggregate data on total health facility visits in hospitals and THCs in Henan Province from January, 2014, to December, 2024, were obtained from the health statistics system. The observation period was divided into the pre-pandemic period (2014–2019), the pandemic period (2020–2022), and the post-pandemic period (2023–2024). A Quasi-Poisson regression model was trained with data from 2014 to 2018. The residual series from the Quasi-Poisson model was then modelled using an LSTM neural network. Data from 2019 were used as the validation set to compare the predictive performance of the Quasi-Poisson model and the Quasi-Poisson-LSTM hybrid model. The final model was used to generate counterfactual predictions for 2020–2024, and relative differences between observed and predicted values were calculated with 95% confidence intervals (CIs).

**Key results:**

In 2024, hospitals and THCs in Henan Province recorded 423 million health facility visits. During 2014–2019, the EAPC in per-person visits was 5.0% (95% CI 3.8–6.1) overall, with a faster increase in hospitals (6.5%, 5.8–7.3) than in THCs (2.2%, −0.5 to 5.0). Over 2014–2024, the EAPC increased to 4.4% (3.0–5.8) in THCs, whereas it decreased to 4.8% (3.8–5.9) in hospitals. The Quasi-Poisson-LSTM model had lower prediction error than the Quasi-Poisson model alone for total visits (RMSE 42.7081 vs. 51.7871; MAE 34.2020 vs. 39.8669). Counterfactual predictions showed that total visits were 14.7% (95% CI 13.2–16.2%) lower than expected in 2020, 4.8% (2.8–7.0%) lower in 2021, and 6.8% (4.3–9.3%) lower in 2022. In 2023 and 2024, total visits returned to near-expected levels, with non-significant relative differences of −1.4% (−4.4% to 1.7%) and 2.3% (−1.2% to 6.0%), respectively. However, facility-specific patterns diverged. In 2024, hospital visits remained 6.0% (3.9–9.5%) below expected levels, whereas THCs visits were 39.7% (20.8–45.0) above expected levels.

**Conclusion:**

The COVID-19 pandemic had divergent effects on health facility visits in hospitals and THCs in Henan Province. Although total visit volume had returned to the expected level in the post-pandemic era, hospital-based visits remained persistently below expected levels, while THCs showed substantial excess use. These findings suggest that care-seeking patterns might have shifted structurally in the post-pandemic era.

## Introduction

The COVID-19 pandemic was one of the most severe public health crises of the 21st century. It caused substantial direct mortality and also affected population health through disruption of routine health services (1). Global estimates suggest that excess mortality associated with the pandemic reached approximately 18.2 million deaths in 2020–2021 (2). The indirect effects of the pandemic were driven by several mechanisms. Health systems faced pressure from the allocation of staff, beds, and other resources to pandemic response (3). Patients delayed or avoided care because of fear of infection. Health facilities suspended or reduced some routine services (4, 5). Mobility restrictions and infection-control requirements further limited access to outpatient and inpatient care (6, 7). Health service utilisation is therefore a sensitive indicator of the wider health-system consequences of COVID-19. Total health facility visits capture changes in population demand for care, access to medical institutions, and the ability of the health system to maintain routine services during public health emergencies (8, 9). Declines in health facility visits might indicate reduced need for some conditions, but they can also indicate unmet need, delayed care, and potential future disease burden (7). Understanding whether service use returned to expected levels after the pandemic is essential for assessing the pace and quality of health-system recovery.

Existing evidence has shown large reductions in health care utilisation during the early phase of the pandemic. Studies from China and other countries reported reductions in outpatient visits, admissions, elective procedures, and selected disease-specific services (10–12). However, most studies in China have focused on the initial outbreak in 2020 or on trends up to 2021. Less is known about the longer-term impact after 3 years of strict pandemic control and after the policy adjustment at the end of 2022. The “New Ten Measures” introduced in late 2022 marked a major change in China’s COVID-19 response (13). After this shift, health care demand, patient behaviour, and pressure on health facilities might have changed substantially (14, 15). Whether health facility visits simply returned to pre-pandemic trajectories, showed compensatory rebound, or shifted between facility types remains uncertain.

This question is especially important in China because the health system has been promoting hierarchical diagnosis and treatment. Under this policy, primary health care facilities are expected to provide first-contact care, chronic disease follow-up, and referral coordination, while hospitals focus more on specialist and severe conditions (16, 17). Township health centres are central to this rural service delivery model. They serve as the hub of the county-township-village health care network and are responsible for basic medical care and public health services in rural communities (18). COVID-19 might have affected hospitals and township health centres (THCs) differently (19). Hospitals may have been more exposed to mobility restrictions, infection-control pressure, and delayed elective care. Township health centres may have maintained more localised basic services and might have absorbed demand redirected from hospitals.

Henan Province provides an informative setting for examining these issues. It is a populous central province with high demand for medical services. In 2024, total health facility visits in the province reached 687 million, indicating a large and active service delivery system. The province also has a large rural population, which makes it suitable for studying the role of THCs in post-pandemic service recovery. We therefore developed a counterfactual prediction framework using monthly health facility visit data from 2014 to 2024. We aimed to assess the long-term impact of COVID-19 on health service utilisation in hospitals and THCs in Henan Province, and to characterise whether recovery differed between facility types after the end of strict pandemic control.

## Methods

### Data sources

We obtained monthly data on total health facility visits in hospitals and THCs in Henan Province from the Center for Health Statistics and Information of the National Health Commission of China. The data covered January, 2014, to December, 2024. Total health facility visits were defined as the total number of person times for all diagnostic activities provided by medical and health institutions. The indicator included outpatient visits, emergency visits, outreach visits, appointment-based consultations, single-item health examinations, and health consultation or guidance visits. Health lectures were excluded. If a patient registered more than once during a single episode of care, visits were counted according to the actual number of diagnostic encounters. The indicator did not include examinations, treatments, or procedures performed according to medical orders, nor did it include immunisation services or health management services. Annual covariate data were extracted from the Henan Health Statistical Yearbook. These covariates included the proportion of the population aged 65 years or older, per-capita GDP, the number of medical and health institutions, and the resident population for each year from 2014 to 2024.

### Statistical analysis

We divided the observation period into three phases: the pre-pandemic period (2014–2019), the pandemic period (2020–2022), and the post-pandemic period (2023–2024). We first calculated Estimated annual percentage changes (EAPCs) in per-person visit rates for hospitals and THCs for 2014–2019, 2014–2022, and 2014–2024. EAPCs were estimated using log-linear regression models, with calendar year as the independent variable and the natural logarithm of total health facility visits divided by resident population as the dependent variable.

To assess the impact of COVID-19 on health facility visits, we constructed a counterfactual prediction model. The pre-pandemic data were divided into a training set and a validation set. Data from 2014 to 2018 were used for model training, and data from 2019 were used for validation. The dependent variable was the monthly number of total health facility visits in hospitals and THCs. Because visit counts were count data and preliminary analyses showed overdispersion, a Quasi-Poisson regression model was used to fit pre-pandemic trends. The model included a continuous time trend, calendar-month indicator variables, the proportion of the population aged 65 years or older, and the natural logarithm of per-capita GDP. These variables adjusted for long-term trends, seasonality, population ageing, and socioeconomic development. The natural logarithms of annual resident population and the number of medical and health institutions were included as offset terms to account for changes in population size and health service supply. The model can be written as:

$$\begin{array}{l} \log(Y_{t})=\beta_{0}+\beta_{1}\times T+\beta_{2}\sum_{M=2}^{12}M+\beta_{3}\times \log(\mathrm{age})\\ +\beta_{4}\times \log(\mathrm{GDP})+\text{offset}(\log(\text{popu})+\log(n)) \end{array}$$

Where *Y_t_* denotes the number of monthly health facility visits; *T* denotes the number of months since January, 2014; *M* denotes calendar-month indicators; age denotes the proportion of the population aged 65 years or older; GDP denotes per-capita GDP; popu denotes resident population; and *n* denotes the number of medical and health institutions.

Although the Quasi-Poisson model can address overdispersion and estimate linear associations between covariates and visit counts, the time series might still contain non-linear patterns, complex seasonal structure, and serial dependence that are not fully captured by a generalised linear model. We therefore modelled the residual series from the Quasi-Poisson regression using a long short-term memory (LSTM) network to extract remaining predictive information. The LSTM model uses input, forget, and output gates to capture long-term dependence in time series data and can complement traditional statistical models by learning non-linear temporal fluctuations.

The residual series from the Quasi-Poisson model was standardised and modelled with a single-layer LSTM (64 hidden units) followed by a dense output layer, using a lookback window of 3 months, the Adam optimizer (learning rate 0.01/0.001), mean squared error loss, batch size 8/16, and 50/100 epochs. Hyperparameters were selected by expanding-window cross-validation on the pre-pandemic data. The predicted residuals from the LSTM model were then added to predictions from the Quasi-Poisson model to obtain the final predictions from the Quasi-Poisson-LSTM hybrid model.

In the validation set, RMSE and MAE were calculated for the Quasi-Poisson model and the Quasi-Poisson-LSTM hybrid model. The model with lower RMSE and MAE was selected as the final prediction model. The selected model was then refitted using all pre-pandemic data from 2014 to 2019 and used to generate counterfactual predictions for monthly health facility visits from 2020 to 2024. These counterfactual predictions represented the expected number of visits if the COVID-19 pandemic had not occurred and if pre-pandemic trends had continued.

Observed values were compared with counterfactual predictions. Relative differences were calculated as (observed value-predicted value)/predicted value. This metric quantified the magnitude of disruption and recovery in health service utilisation in hospitals and THCs. The 95% CIs were calculated using a parametric bootstrap based on the multivariate normal distribution of model coefficients, with 1,000 simulations.

We assessed the robustness of our findings in four sensitivity analyses. First, we reduced the number of LSTM units to 32. Second, we fitted an ARIMA–LSTM hybrid, comprising an ARIMA model of the first-differenced log-transformed series with seasonal and autoregressive terms, followed by an LSTM model of the ARIMA residuals. Third, we conducted an interrupted time-series analysis using segmented quasi-Poisson regression, with the same covariates and offsets as the main model and interruptions in January, 2020, and January, 2023. Counterfactual predictions were obtained by setting the pandemic-related segment terms to zero, assuming that the pre-pandemic trend and seasonal pattern would have continued. Fourth, we included the provincial proportion of tertiary hospitals as an additional covariate.

All analyses were conducted in R software, version 4.2.1. LSTM models were fitted using the keras3 and tensorflow packages. Bootstrapping was conducted using the boot package. Statistical significance was set at *α* = 0.05.

## Results

Total health facility visits in hospitals and THCs in Henan Province increased from 255.3 million in 2014 to 422.8 million in 2024 (Table 1). Hospital visits increased from 158.8 million to 264.8 million, and visits to THCs increased from 96.5 million to 158.0 million. Trend analysis based on per-person visits showed that, during the pre-pandemic period (2014–2019), the EAPC was 5.0% (95% CI 3.8–6.1) overall (Table 2). The increase was more pronounced in hospitals, with an EAPC of 6.5% (5.8–7.3). In THCs, the EAPC was 2.2% (−0.5 to 5.0) and was not statistically significant. When the observation period was extended to 2022, the overall EAPC decreased to 3.9% (2.8–5.0), and the hospital EAPC decreased to 4.4% (2.9–6.0), whereas the EAPC in THCs increased to 3.1% (1.7–4.4). After 2023–2024 were included, the EAPC for 2014–2024 was 4.6% (3.7–5.6) overall, 4.8% (3.8–5.9) in hospitals, and 4.4% (3.0–5.8) in THCs. Compared with 2014–2022, growth in per-person visits strengthened overall and in THCs.

**Table 1:** Annual total health facility visits in hospitals and THCs in Henan Province, 2014–2024.

Year	All visits (10,000)	Visits per person	Hospital visits (10,000)	Visits per person	THC visits (10,000)	Visits per person
2014	25,532.3	2.65	15,883.6	1.65	9,648.7	1.00
2015	27,323.7	2.82	16,867.3	1.74	10,456.4	1.08
2016	29,364.3	3	18,145.8	1.86	11,218.5	1.15
2017	29,772.0	3.03	19,134.8	1.95	10,637.3	1.08
2018	31,545.3	3.2	20,711.9	2.1	10,833.4	1.1
2019	34,005.7	3.43	22,450.7	2.27	11,554.9	1.17
2020	31,741.1	3.19	20,277.7	2.04	11,463.4	1.15
2021	35,801.7	3.62	23,160.8	2.34	12,640.9	1.28
2022	36,193.2	3.67	22,852.1	2.31	13,341.1	1.35
2023	40,994.9	4.18	25,769.4	2.63	15,225.6	1.55
2024	42,275.3	4.32	26,479.9	2.71	15,795.4	1.61

**Table 2:** Temporal trends in per-person health facility visit in hospitals and THCs in Henan Province, 2014–2024.

Period	All, %	Hospital, %	THC, %
2014–2019	5.0 (3.8, 6.1)	6.5 (5.8, 7.3)	2.2 (−0.5, 5.0)
2014–2022	3.9 (2.8, 5.0)	4.4 (2.9, 6.0)	3.1 (1.7, 4.4)
2014–2024	4.6 (3.7, 5.6)	4.8 (3.8, 5.9)	4.4 (3.0, 5.8)

In expanding-window cross-validation of the LSTM residual models, the optimal hyperparameter combination for total visits was a learning rate of 0.01, 50 epochs, and a batch size of 8 (RMSE = 0.3815, MAE = 0.2408). For hospitals, the optimal combination was a learning rate of 0.01, 100 epochs, and a batch size of 8 (RMSE = 0.7906, MAE = 0.5840). For THCs, the optimal combination was a learning rate of 0.01, 50 epochs, and a batch size of 8 (RMSE = 0.1932, MAE = 0.1584; Table 3). In the validation set, the Quasi-Poisson-LSTM hybrid model had lower prediction errors than the Quasi-Poisson model alone. For total visits, RMSE decreased from 51.7871 to 42.7081, and MAE decreased from 39.8669 to 34.2020. For hospitals, RMSE decreased from 53.5979 to 52.2672, and MAE decreased from 46.1588 to 44.1918. For THCs, RMSE decreased from 54.7325 to 45.7839, and MAE decreased from 41.0997 to 37.0362. We therefore selected the Quasi-Poisson-LSTM hybrid model as the final prediction model (Table 4).

**Table 3:** Expanding-window cross-validation results for the LSTM residual models.

Facility	Learning rate	Batch	Epochs	RMSE	MAE
All	0.01	8	50	0.3815	0.2408
	0.01	8	100	0.6438	0.4654
	0.01	16	50	0.5405	0.4437
	0.01	16	100	0.5001	0.4729
	0.001	8	50	0.5785	0.5160
	0.001	8	100	0.6066	0.4282
	0.001	16	50	0.6126	0.5568
	0.001	16	100	0.6088	0.4548
Hospitals	0.01	8	50	1.1707	0.9445
	0.01	8	100	0.7906	0.5840
	0.01	16	50	1.2996	1.1880
	0.01	16	100	0.8743	0.6687
	0.001	8	50	0.8344	0.6457
	0.001	8	100	0.8261	0.6655
	0.001	16	50	0.8524	0.6808
	0.001	16	100	0.8154	0.6672
THCs	0.01	8	50	0.1932	0.1584
	0.01	8	100	0.2650	0.1953
	0.01	16	50	0.2331	0.2023
	0.01	16	100	0.6753	0.6034
	0.001	8	50	0.3658	0.3198
	0.001	8	100	0.5374	0.5166
	0.001	16	50	0.2862	0.2596
	0.001	16	100	0.5398	0.5200

**Table 4:** Comparison of the Quasi-Poisson model and Quasi-Poisson-LSTM hybrid model in the validation set.

Facility	Model	RMSE	MAE
All	Quasi-Poisson	51.7871	39.8669
	Quasi-Poisson-LSTM	42.7081	34.2020
Hospitals	Quasi-Poisson	53.5979	46.1588
	Quasi-Poisson-LSTM	52.2672	44.1918
THCs	Quasi-Poisson	54.7325	41.0997
	Quasi-Poisson-LSTM	45.7839	37.0362

Comparisons between observed and counterfactual values showed that the pandemic caused a substantial disruption to health service utilisation in 2020–2022 (Figure 1), with marked heterogeneity between hospitals and THCs in each year from 2020 to 2024 (all *P*_difference_ < 0·001; Table 5). In 2020, total visits were 14.7% (95% CI 13.2–16.2%) lower than expected. Hospital visits were 21.3% (20.2–22.6%) lower than expected. In 2021 and 2022, total visits remained below counterfactual expectations, with reductions of 4.8% (2.8–7.0%) and 6.8% (4.3–9.3%), respectively. Hospital visits remained significantly below expected levels in 2021 and 2022, with reductions of 11.2% (10.2–13.5%) and 13.9% (12.6–16.4%). By contrast, visits to THCs were 14.2% (1.5–14.1) above expected in 2021 and 17.5% (3.4–18.8%) above expected in 2022. After the adjustment of COVID-19 control policies at the end of 2022, total health facility visits in Henan Province rebounded in 2023 and 2024. The differences from counterfactual expectations were not statistically significant, at −1.4% (−4.4% to 1.7%) in 2023 and 2.3% (−1.2% to 6.0%) in 2024, suggesting that overall health service utilisation had returned to the level expected from pre-pandemic trends. However, divergent facility-specific patterns were observed. Hospital visits remained persistently below expected levels, with reductions of 11.6% (9.6–14.3%) in 2023 and 6.0% (3.9–9.5%) in 2024. In contrast, visits to THCs were significantly above expected levels, increasing by 24.6% (17.3–37.9%) in 2023 and 39.7% (20.8–45.0) in 2024.

**Figure 1:**
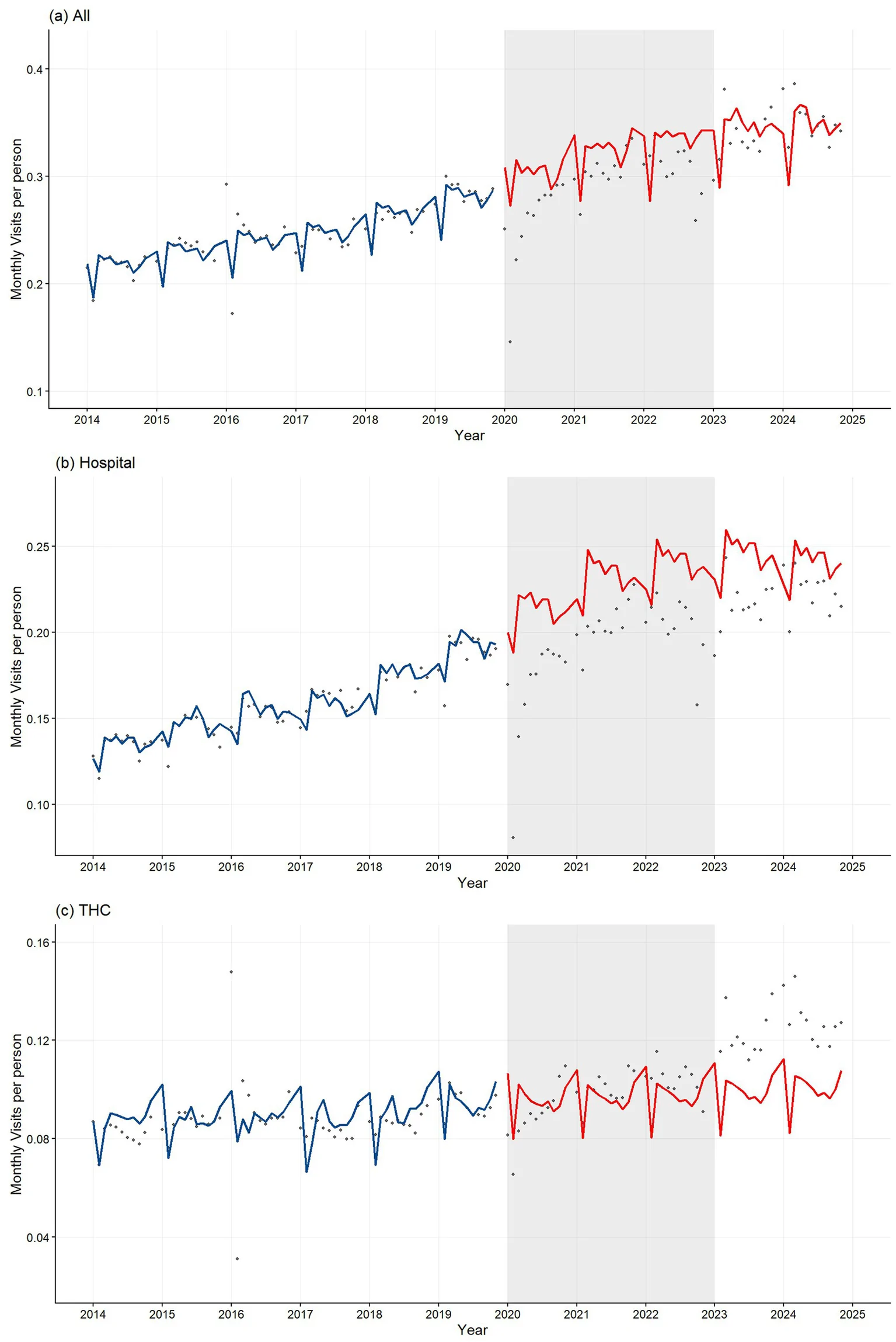
Monthly per-person health facility visits in hospitals and THCs in Henan Province, 2014–2024. Black dots show observed monthly per-person visits. The blue line shows fitted values from the Quasi-Poisson-LSTM hybrid model. The red line shows counterfactual predictions from the Quasi-Poisson-LSTM hybrid model under the assumption that the COVID-19 pandemic did not occur. **(a)** All, **(b)** hospital, **(c)** THC.

**Table 5:** Relative changes in health facility visits per person during and after the COVID-19 pandemic by facility type (%).

Year	All, % (95% CI)	Hospital, % (95% CI)	THC, % (95% CI)	*P_difference_*
2020	−14.7 (−16.2, −13.2)	−21.3 (−22.6, −20.2)	−2.5 (−6.8, 2.5)	<0.001
2021	−4.8 (−7.0, −2.8)	−11.2 (−13.5, −10.2)	14.2 (1.5, 14.1)	<0.001
2022	−6.8 (−9.3, −4.3)	−13.9 (−16.4, −12.6)	17.5 (3.4, 18.8)	<0.001
2023	−1.4 (−4.4, 1.7)	−11.6 (−14.3, −9.6)	34.6 (17.3, 37.9)	<0.001
2024	2.3 (−1.2, 6.0)	−6.0 (−9.5, −3.9)	39.7 (20.8, 45.0)	<0.001

Findings were consistent across all four sensitivity analyses (Supplementary Tables S1–S4). Estimated counterfactual visits per person changed little compared with the main analysis. The overall pattern remained unchanged. Total visits were approximately at expected levels in 2023–2024, whereas hospital visits remained below expected levels and township health-centre visits were above expected levels.

## Discussion

This study used monthly total health facility visit data from Henan Province from 2014 to 2024 and applied a Quasi-Poisson-LSTM hybrid model to construct a counterfactual prediction framework. We systematically assessed the long-term trend of health facility visits in hospitals and THCs and the impact of the COVID-19 pandemic. Health service utilisation in Henan increased steadily before the pandemic, but the early phase of COVID-19 in 2020 caused a substantial disruption to routine services. Total visits were 15.3% lower than expected in 2020. The reduction was much larger in hospitals (19.0%) than in THCs (5.9%). After the adjustment of COVID-19 control policies at the end of 2022, overall visit volume returned to the expected level in 2023–2024. However, hospital visits remained persistently below expected levels, while visits to THCs were significantly above expected levels. These findings suggest that the recovery of health service utilisation in Henan was not a simple return to the pre-pandemic trajectory. It might have been accompanied by a change in the structure of care-seeking behaviour.

The marked reduction in visit volume in Henan during the early pandemic period is consistent with several national studies. A time series analysis in China reported significant decreases in outpatient and emergency service use across facility types in early 2020, with heterogeneous effects across regions and institutions (8). A study based on the China Health Statistical Yearbook also reported decreases in national outpatient visits and admissions in 2020, and showed that the magnitude of disruption differed by hospital type, department, and region (12). Another interrupted time series study using national routine health information system data showed that the 2022 Omicron outbreak and associated control measures in Shanghai led to a cumulative reduction of 23.5 million outpatient visits in Shanghai and 150.3 million outpatient visits in other regions (20). The decline in visits during the early pandemic can be explained by both demand-side and supply-side factors. On the demand side, mobility restrictions, restrictions on gatherings, and fear of infection in health care settings reduced willingness to seek care, especially for non-urgent and elective services (4, 21). Henan is a populous province in central China, and high quality medical resources are concentrated in secondary and tertiary hospitals. Cross city care seeking is common. During the pandemic, traffic control and restrictions on population movement reduced access to hospitals and led to a marked decline in hospital visits. On the supply side, health facilities might have suspended some non-emergency services and shifted staff, beds, and other resources towards pandemic control, thereby reducing the supply of routine care (22). The fact that total visits remained 5.9 and 7.3% below expected levels in 2021 and 2022 indicates that the impact of the pandemic was sustained and was not limited to the initial shock in 2020.

We found that total visits in Henan had broadly returned to expected levels in 2023 and 2024. However, hospital and township health-centre visits showed opposite patterns. Visits to THCs were significantly above expected levels in the post-pandemic period, whereas hospital visits remained significantly below expected levels. This pattern might have two explanations. First, after the policy adjustment in December, 2022, primary health care facilities took on a larger role in first-contact care and management of common conditions. Residents might have shifted towards nearby facilities. A cross-sectional survey in Beijing reported that many patients selected primary health care institutions as the first point of care during the period of increased SARS-CoV-2 infections (23). Another study of infected individuals in Beijing in December, 2022, reported that more than 70% of those seeking treatment chose primary health care institutions (19). These findings are consistent with the direction observed in Henan, where THCs showed a significant increase in visits after the policy adjustment. Second, China’s hierarchical diagnosis and treatment system might have further strengthened the role of primary health care facilities after the pandemic. The policy encourages first contact at the primary-care level, two-way referral, and diversion of patients from large hospitals, with the aim of reducing pressure on hospitals and improving the use of primary care (24). Local diagnosis and treatment pathways also clarified the functions of different institutions. For example, the 2022 Shanghai pathway assigned primary health care institutions responsibility for the initial identification and classification of COVID-19 infections, standardised treatment and health monitoring for stable patients, and timely referral for high-risk or deteriorating patients. Higher-level hospitals supported lower-level institutions through medical alliances and remote consultation. When such pathways became operational, some demand that would previously have flowed directly to hospitals might have been assessed and managed first in THCs. This shift could explain the increase in township health-centre visits at the population level. However, the policy context and studies cited above support the plausibility of these explanations rather than establish the mechanisms underlying the patterns observed in Henan. Greater use of THCs may reflect changes in where patients seek care, but it may also arise from additional healthcare needs or repeated visits. In particular, persistent symptoms after SARS-CoV-2 infection can require assessment and follow-up in primary care (25). Such encounters could increase THC visit counts without replacing hospital visits. Changes in data collection or reporting may also influence recorded trends. Research across 10 countries identified disruption to routine health information reporting during the pandemic as a potential source of bias in analyses of facility-based service counts (3). We could not assess the contribution of these factors in Henan. Our aggregate data contain no information on patient identity, referral pathways, or movement between facilities. We therefore interpret the findings as divergent facility-specific visit trajectories and cannot determine whether the increase in THC visits reflects additional demand, repeated encounters, or substitution for hospital care.

The Quasi-Poisson-LSTM hybrid model used in this study has methodological advantages over some conventional time series approaches. Poisson, ARIMA models and other generalised linear models estimate counterfactual trends by imposing assumptions about the functional form of the pre-pandemic time series. They may not fully capture complex non-linear dependence in health service utilisation data (26, 27). In this study, LSTM was used to model the residuals from the Quasi-Poisson regression. This approach allowed the model to learn temporal dependence in the residual series without specifying a fixed sequence structure. It therefore improved the accuracy of counterfactual prediction. Chen and colleagues used a similar ARIMA-LSTM hybrid strategy to assess the impact of the COVID-19 pandemic on disease burden across 204 countries and territories, and reported better predictive performance than ARIMA alone (28). Our findings support the use of hybrid modelling approaches for health service utilisation forecasting, especially when only provincial-level routine data are available.

The post-pandemic increase in township health-centre visits has policy implications. National health statistics show that primary health care institutions now provide a large proportion of total health facility visits in China. The significant increase in township health-centre visits observed in Henan in 2023–2024 suggests that primary health care facilities might have taken on a larger share of basic diagnostic and health service demand in the post-pandemic period. Local health policy should therefore focus not only on whether total visit volume has recovered, but also on changes in service function, resource allocation, and patient flow between hospitals and primary care. Rapid growth in primary-care use could increase pressure on staff, equipment, drug supply, and service quality if capacity does not expand accordingly.

This study has several limitations. First, Henan is a populous province in central China, and its urban–rural structure, socioeconomic level, and distribution of medical resources differ from those of eastern coastal provinces and less developed western provinces. The findings might therefore not be directly generalisable to all provinces in China. Second, total health facility visits are an aggregate indicator. We could not distinguish first visits from follow-up visits, acute from chronic conditions, or different clinical departments. This limited our ability to examine changes in disease composition behind the observed visit trends. Third, this study included only hospitals and THCs. Community health service centres, village clinics, outpatient departments, and other facilities were not included. Changes in some primary-care services might therefore have been underestimated or missed. Fourth, The LSTM component was trained on only 60 monthly observations from 2014 to 2018, with fewer usable training examples after constructing lagged sequences. This limited sample may increase the risk of overfitting and reduce model stability.

## Conclusion

COVID-19 substantially disrupted health facility visits in Henan Province, especially in hospitals. After the end of strict pandemic control, total visit volume returned to expected levels, but hospital visits remained below expected levels while township health-centre visits increased substantially. These findings suggest a post-pandemic shift in care-seeking towards rural primary care, which should be supported through stronger township-level capacity, better resource allocation, and improved referral coordination under China’s hierarchical diagnosis and treatment system.

## Data Availability

The original contributions presented in the study are included in the article/Supplementary material, further inquiries can be directed to the corresponding author.
