# Supplementary material for: Unequal recovery of health facility visits in hospitals and township health centres after COVID-19 disruption in Henan Province, China, 2014–2024: a time-series study
Source: Front Public Health. 2026 Oct 28;14:1916015. doi: 10.3389/fpubh.2026.1916015 (PMC13752064; doi:10.3389/fpubh.2026.1916015)
Supplement: Supplementary file 1 [file Table_1.DOCX]

Table S1. Comparison of estimates from Quasi-Poisson–LSTM models with 64 and 32 LSTM units

| Facility | Neurons | Year | Change (%) | 95% CI |
| --- | --- | --- | --- | --- |
| Total | 64 | 2020 | -14.7 | (-16.2, -13.2) |
|  |  | 2021 | -4.8 | (-7.0, -2.8) |
|  |  | 2022 | -6.8 | (-9.3, -4.3) |
|  |  | 2023 | -1.4 | (-4.4, 1.7) |
|  |  | 2024 | 2.3 | (-1.2, 6.0) |
|  | 32 | 2020 | -14.7 | (-16.2, -13.1) |
|  |  | 2021 | -4.8 | (-6.9, -2.7) |
|  |  | 2022 | -6.8 | (-9.2, -4.2) |
|  |  | 2023 | -1.4 | (-4.3, 1.8) |
|  |  | 2024 | 2.3 | (-1.2, 6.1) |
| Hospital | 64 | 2020 | -21.3 | (-22.6, -20.2) |
|  |  | 2021 | -11.2 | (-13.5, -10.2) |
|  |  | 2022 | -13.9 | (-16.4, -12.6) |
|  |  | 2023 | -11.6 | (-14.3, -9.6) |
|  |  | 2024 | -6.0 | (-9.5, -3.9) |
|  | 32 | 2020 | -21.3 | (-22.6, -20.2) |
|  |  | 2021 | -11.4 | (-13.3, -9.9) |
|  |  | 2022 | -14.1 | (-16.2, -12.3) |
|  |  | 2023 | -11.2 | (-13.7, -9.1) |
|  |  | 2024 | -6.2 | (-9.2, -3.6) |
| THC | 64 | 2020 | -2.5 | (-6.8, 2.5) |
|  |  | 2021 | 14.2 | (1.5, 14.1) |
|  |  | 2022 | 17.5 | (3.4, 18.8) |
|  |  | 2023 | 34.6 | (17.3, 37.9) |
|  |  | 2024 | 39.7 | (20.8, 45.0) |
|  | 32 | 2020 | -2.5 | (-7.2, 2.4) |
|  |  | 2021 | 11.5 | (5.0, 18.2) |
|  |  | 2022 | 14.5 | (6.9, 23.2) |
|  |  | 2023 | 31.2 | (21.3, 42.8) |
|  |  | 2024 | 36.2 | (24.4, 50.3) |

Table S2. Comparison of estimates from the Quasi-Poisson–LSTM and ARIMA–LSTM models

| Facility | Model | Year | Change (%) | 95% CI |
| --- | --- | --- | --- | --- |
| Total | QP-LSTM | 2020 | -14.7 | (-16.2, -13.2) |
|  |  | 2021 | -4.8 | (-7.0, -2.8) |
|  |  | 2022 | -6.8 | (-9.3, -4.3) |
|  |  | 2023 | -1.4 | (-4.4, 1.7) |
|  |  | 2024 | 2.3 | (-1.2, 6.0) |
|  | ARIMA-LSTM | 2020 | -12.1 | (-13.7, -10.5) |
|  |  | 2021 | -2.0 | (-4.1, 0.2) |
|  |  | 2022 | -4.7 | (-7.2, -2.1) |
|  |  | 2023 | -3.8 | (-6.7, -0.7) |
|  |  | 2024 | -5.0 | (-8.1, -1.4) |
| Hospital | QP-LSTM( | 2020 | -21.3 | (-22.6, -20.2) |
|  |  | 2021 | -11.2 | (-13.5, -10.2) |
|  |  | 2022 | -13.9 | (-16.4, -12.6) |
|  |  | 2023 | -11.6 | (-14.3, -9.6) |
|  |  | 2024 | -6.0 | (-9.5, -3.9) |
|  | ARIMA-LSTM | 2020 | -16.7 | (-18.1, -15.5) |
|  |  | 2021 | -4.0 | (-6.0, -2.4) |
|  |  | 2022 | -11.5 | (-13.7, -9.7) |
|  |  | 2023 | -13.7 | (-16.1, -11.6) |
|  |  | 2024 | -17.1 | (-19.8, -14.8) |
| THC | QP-LSTM | 2020 | -2.5 | (-6.8, 2.5) |
|  |  | 2021 | 14.2 | (1.5, 14.1) |
|  |  | 2022 | 17.5 | (3.4, 18.8) |
|  |  | 2023 | 34.6 | (17.3, 37.9) |
|  |  | 2024 | 39.7 | (20.8, 45.0) |
|  | ARIMA-LSTM | 2020 | -5.3 | (-9.9, -0.6) |
|  |  | 2021 | 0.4 | (-5.4, 6.5) |
|  |  | 2022 | 1.8 | (-5.0, 9.4) |
|  |  | 2023 | 14.9 | (6.2, 25.1) |
|  |  | 2024 | 18.2 | (8.0, 30.4) |

Table S3. Interrupted time series (ITS) analysis of relative changes in health facility visits per person during and after the COVID-19 pandemic by facility type

| Year | All, % (95% CI) | Hospital, % (95% CI) | THC, % (95% CI) | P_difference_ |
| --- | --- | --- | --- | --- |
| 2020 | -14.7  (-16.1, -13.1) | -21.3  (-22.9, -19.9) | -2.4  (-5.2, 0.7) | <0.001 |
| 2021 | -5.1  (-7.0, -2.9) | -11.8  (-13.9, -9.9) | 7.3  (3.6, 11.6) | <0.001 |
| 2022 | -7.0  (-9.3, -4.4) | -14.4  (-16.9, -12.2) | 10.3  (5.8, 15.6) | <0.001 |
| 2023 | -1.6  (-4.4, 1.7) | -11.9  (-14.8, -9.2) | 36.4  (20.3, 33.8) | <0.001 |
| 2024 | 2.0  (-1.4, 6.0) | -6.6  (-10.1, -3.3) | 31.3  (23.9, 40.4) | <0.001 |

Table S4. Comparison of estimates from the main model and the model additionally adjusted for the proportion of tertiary hospitals

| Model | 2020 | 2021 | 2022 | 2023 | 2024 |
| --- | --- | --- | --- | --- | --- |
| Model 1* | -21.3 | -11.7 | -14.3 | -11.8 | -6.5 |
| Model 2** | -21.7 | -12.8 | -16.7 | -15.2 | -10.3 |

** Main model, * adjusted for the proportion of tertiary hospitals
